# Skeleton Genetics: a comprehensive database for genes and mutations related to genetic skeletal disorders

**DOI:** 10.1093/database/baw127

**Published:** 2016-08-31

**Authors:** Chong Chen, Yi Jiang, Chenyang Xu, Xinting Liu, Lin Hu, Yanbao Xiang, Qingshuang Chen, Denghui Chen, Huanzheng Li, Xueqin Xu, Shaohua Tang

**Affiliations:** 1 Department of Genetics of Dingli Clinical Medical School, Wenzhou Central Hospital, Wenzhou 325000, China; 2 Institute of Genomic Medicine, Wenzhou Medical University, Wenzhou 325035, China; 3 School of Laboratory Medicine and Life Science, Wenzhou Medical University, Wenzhou 325035, China

## Abstract

Genetic skeletal disorders (GSD) involving the skeletal system arises through disturbances in the complex processes of skeletal development, growth and homeostasis and remain a diagnostic challenge because of their clinical heterogeneity and genetic variety. Over the past decades, tremendous effort platforms have been made to explore the complex heterogeneity, and massive new genes and mutations have been identified in different GSD, but the information supplied by literature is still limited and it is hard to meet the further needs of scientists and clinicians. In this study, combined with Nosology and Classification of genetic skeletal disorders, we developed the first comprehensive and annotated genetic skeletal disorders database, named ‘SkeletonGenetics’, which contains information about all GSD-related knowledge including 8225 mutations in 357 genes, with detailed information associated with 481 clinical diseases (2260 clinical phenotype) classified in 42 groups defined by molecular, biochemical and/or radiographic criteria from 1698 publications. Further annotations were performed to each entry including Gene Ontology, pathways analysis, protein–protein interaction, mutation annotations, disease–disease clustering and gene–disease networking. Furthermore, using concise search methods, intuitive graphical displays, convenient browsing functions and constantly updatable features, ‘SkeletonGenetics’ could serve as a central and integrative database for unveiling the genetic and pathways pre-dispositions of GSD.

**Database URL:** http://101.200.211.232/skeletongenetics/

## Introduction

The skeleton provides the structural framework in humans for muscle attachments, assists movement, protects organs, and maintains the homeostasis of the vascular systems (1). Perturbation of development in bone, cartilage and joints could result in human skeletal dysplasias, which affect approximately 1 in 5000 live births and is one of the common causes of neonatal birth defect in departments of obstetrics (2). Genetic skeletal disorders (GSD), which are a group of disorders involving gene mutations or genetic susceptibility to bone disease, account for most of the human skeletal dysplasias, involving a variety of pathogenic factors and diseases (3). In the *Nosology and Classification of Genetic Skeletal Disorders 2015 revision* (hereinafter referred to as NCGSD-2015), 436 conditions were included and placed in 42 groups, which were associated with mutations in one or more of 336 unique genes (4). Till now, especially with the impact the next-generation sequencing technology has made on the study of genetic skeletal disorders, a large amount of mutations and novel GSD-related genes have been identified. Genetic skeletal disorders face similar problems to other complex diseases with exceptional genetic heterogeneity and clinical variety (5). Notably, the same skeletal dysplasia gene can lead to substantially different clinical phenotypes or a specific skeletal phenotype can be caused by mutations in different genes (6). Therefore, understanding genotype–phenotype correlations and functional diversity remains one of the major challenges for GSD.

The NCGSD-2015 provides an overview of the recognized diagnostic entities of GSD by clinical, anatomical site and molecular pathogenesis for clinicians, scientists and the radiology community to diagnose individual cases and describe novel disorders (5). However, the increasing availability of next-generation sequencing technology and other new sequencing platforms will likely result in a rapid identification of novel GSD-related genes and mutations, and novel phenotypes associated with mutations in genes linked to other phenotypes has increased dramatically to date (7, 8). Therefore, the catalog of GSD-related genotype-phenotype has become so large as to surpass the scope of a ‘Nosology’, so the Nosology must be transformed into an automated annotation and query database. Thus, it is crucial to integrate the existing data and present an organized comprehensive mutation repository to construct an integrative, informative and updatable resource for GSD-related genetic pre-dispositions which could greatly facilitate the counseling, diagnosis and therapy for pediatrics and genetics.

In this study, we reviewed three dependent databases and public resources related to genetic skeletal disorders and made the first annotated database about GSD, named ‘SkeletonGenetics’, which provides full-scale gene and mutation information and extensive annotations for GSD. Initially, we retrospectively extracted the basic information for each mutation (disease conditions or syndrome, gene symbol, number of OMIM, hereditary mode, coding sequence change, transcript, ethnicity, age, PubMed ID, etc.) from open publications. Additionally, extensive annotations were performed, which include gene information, Gene Ontology, pathways analysis, protein-protein interaction, mutations clustering and gene-disease network. As a result, 42 groups of GSD, 481 diseases or syndromes, 357 genes, 5884 single nucleotide variations (SNVs), 516 insertions, 1427 deletions and 2260 different phenotypes were included. Taken together, ‘SkeletonGenetics’ is constructed to be a well-organized, internal-standard and comprehensive resource for scientists and clinicians looking for the clinical correlates of mutations, genes and pathways involved in skeletal biology.

## Methods

### The standard of data entry group of GSD

The Nosology Group of the International Skeletal Dysplasia Society formulated the table of NCGSD-2015 in September, 2015. The criteria used for genetic skeletal disorders were unchanged from NCGSD-2010 revision. They were (1) significant skeletal involvement, including skeletal dysplasias, skeletal dysostoses, metabolic bone disorders and skeletal malformation and/or reduction; (2) publication and/or listing in OMIM; (3) genetic basis proven by pedigree or very likely based on homogeneity of phenotype in unrelated families; (4) nosologic autonomy confirmed by molecular or linkage analysis and/or by the presence of distinctive diagnostic features and of observation in multiple individuals or families (4). In accordance with the standards of NCGSD-2015, 481 different conditions were placed in 42 groups, associated with one or more of 357 different genes. The grouping results and criteria are presented in Supplementary Table S1.

### Data collection and content

Based on the standards of NCGSD-2015, we performed comprehensive searches for GSD-related publications and databases to obtain a complete list of mutation information associated with GSD. Firstly, we retrospectively queried the PubMed database (http://www.ncbi.nih.gov/pubmed) for genes and diseases retrieved in Entrez with terms ‘gene or disease symbol [Title/Abstract] AND mutation [Title/Abstract] OR variant [Title/Abstract]. Secondly, other databases were taken as supplementary sources, including Leiden Open Variation Database 3.0 (LOVD3.0, http://www.lovd.nl/3.0/home) which contained fanconi anemia relevant genes and the mutations predicted to be benign or which did not segregate with phenotype were excluded (9), Clinvar (http://www.ncbi.nlm.nih.gov/clinvar/), which contained pathogenic or likely pathogenic clinical mutation information. Human Phenotype Ontology (HPO, http://human-phenotype-ontology.github.io/) (10) and Online Mendelian Inheritance in Man (OMIM) (11) were used to describe phenotypic information. Patient clinical data have been obtained in accordance with the tenets of the Declaration of Helsinki.

Finally, >2000 publications were queried from open resource beginning in 1990. After manually screening the abstracts and full-text of these publications, we excluded those studying diseases other than GSD and eventually retained 1698 publications. We extracted the basic information for each mutation, including disease name, gene symbol, inheritance mode, CDS change, PubMed ID, ethnicity, age, gender and functional study through reading the full-text articles and double-checked these manually. The in-house perl program was used to obtain the correct genomic coordinate for each entry. As of the beginning of 2016, the ‘SkeletonGenetics’ database contains 481 disease conditions, associated with mutations in one or more of 357 different genes, 8225 variations (5884 SNVs, 1427 deletions, 516 insertions and 398 Block substitution), 2260 clinical phenotypes and their detailed information included in 42 groups of GSD from 1698 publications ranging from common, recurrent mutations to ‘private’ mutations found in single families or individuals. The results are presented in Table 1.

**Table 1:** Data content and statistics of genetic data in SkeletonGenetics

Data type	Data count
Mutation	8225
SNVs	5884
Deletions	1427
Insertions	516
Block substitution	398
Genes	357
Disease group	42
Disease or syndrome	481
Phenotypes	2260
GOs	2535
Pathways	138
KEGG pathways	64
Wiki pathways	74
MicroRNA Target	66
PPI	40
Publications	1698

### Information statistics

Specific skeletal phenotype can be caused by mutations in different genes or the same gene can lead to substantially different clinical phenotypes. This leads to the bias of genotype and phenotype of GSD, therefore statistics on the number of mutations for gene, disease and the group become very important. The gene Top 5 with the highest number of mutations in ‘SkeletonGenetics’ is ‘FBN1’, associated with four skeletal diseases (Marfan syndrome, Weill-Marchesani syndrome 2, Geleophysic dysplasia type 2, Acromicric dysplasia) with 639 mutations; followed by ‘NF1’ associated with Neurofibromatosis type 1 with 415 mutations; ‘NIPBL’ associated with Cornelia de Lange syndrome 1 with 406 mutations; ‘NSD1’ associated with Sotos syndrome 1 with 362 mutations; ‘GNPTAB’ associated with two diseases (Mucolipidosis II, alpha/beta type; Mucolipidosis III (Pseudo-Hurler polydystrophy), alpha/beta type) with 231 mutations. Correspondingly, Marfan syndrom, Fanconi anemia, Neurofibromatosis, Sotos syndrome, Cornelia de Lange syndrome, which all belong to the Top 5 of the disease list, all have >367 gene mutation information. At the same time, we classified the number of mutations by group: the limb hypoplasia-reduction defects group; the overgrowth syndromes with skeletal involvement group; the lysosomal storage diseases with skeletal involvement (dysostosis multiplex group); the disorganized development of skeletal components group; and the dysostoses with predominant craniofacial involvement group all have >375 mutation information. Finally, according to the information collected, we statistically analyzed the bias by chromosomes, mutation types, mutation effect, gender, ages of onset and inheritance mode.

### Functional and enrichment analysis

**Figure 1.**
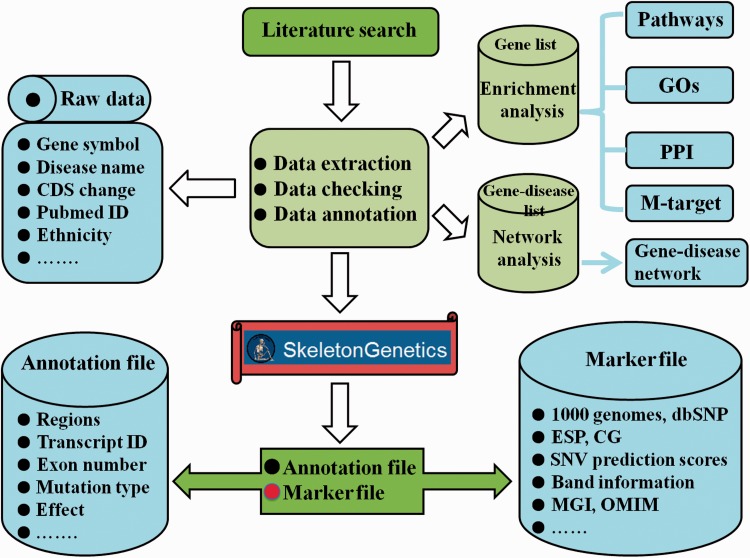
Flowchart of the procedure for ‘SkeletonGenetics’. ‘SkeletonGenetics’ mainly consists of three parts: (i) data extraction based on literature search and GSD-related databases, (ii) annotation of all mutations and genes using ANNOVAR and (iii) enrichment analysis by WebGestalt and gene–disease network analysis graphically.

To further interpret the function and heterogeneity of GSD, ‘SkeletonGenetics’ performs a series of functional analyses, which included enrichment analysis, mutation annotation, mutation spectrum and gene-disease network construction (Figure 1).

For the aspect of enrichment analysis, we provided Gene Ontology (GO), KEGG pathways, Wikipathways, MicroRNA Target and Protein–Protein interaction. Meanwhile, we used WebGestalt software to store the gene list in ‘SkeletonGenetics’ (12, 13). Users can click on the ‘Analysis’ page to see whether the gene of interest is involved in any GO terms, function pathways, microRNA target or PPIs.

Gene ontology annotation terms involved the biological process, the cellular component and molecular function of three biological processes, the Top5 most statistically significant (*P*-values) of biological process terms in ‘SkeletonGenetics’, namely, skeletal system development (*P*-value: 3.29E-63, number of genes, 87) (14); appendage development (*P*-value: 6.25E-46, number of genes, 50) (15); limb development (*P*-value: 6.25E-46, number of genes, 50) (16); appendage morphogenesis (*P*-value: 5.11E-45, number of genes, 48) (15); and limb morphogenesis (*P*-value: 5.11E-45, number of genes, 48) (16). The above entries of the most statistically significant GOs terms are all related to skeletal system or component formation, morphological differentiation and location control.

Meanwhile, we provided functions pathways, including KEGG pathways and Wikipathways of two annotation methods. A complex series of signaling pathways including TGF-beta signaling pathway (*P*-value: 1.07E-13, number of genes, 14) (17); Hedgehog signaling pathway (*P*-value: 5.85E-09, number of genes, 9) (18); WNT signaling pathway and pluripotency (*P*-value: 1.72E-06, number of genes, 10) (19); Notch signaling pathway (*P*-value: 4.79E-07, number of genes, 7) (20) and metabolic processing (*P*-value: 1.48E-14, number of genes, 41) are essential for proper skeletogenesis (21), mainly distributed in the cell, extracellular matrix, and transcriptional regulation related to bone, cartilage, and joint formation. Researchers recommend use of a morphogen rheostat model to conceptualize the differential signaling inputs which lead to divergent skeletal phenotypes within a temporal and spatial context (1). In these terms, we have established some function pathways of the relationship between different gene mutations and groups of bone diseases.

The modification of microRNA by degraded target mRNA maintains cellular homeostasis and regulates cell fate transitions during differentiation. These processes are important to ensure proper organogenesis and growth of skeleton. ‘hsa_CTTTGCA, MIR-527’, which ranks first with the *P*-value of 3.93E-07 among all MicroRNA Target enriched, including the MYCN gene, in which miRNA cluster heterozygous mutations cause Feingold syndrome, a disorder that involves limb malformations, microcephaly, learning disability/mental retardation, hand and foot abnormalities and may include hypoplastic thumbs, clinodactyly of the second and fifth fingers, syndactyly (characteristically between the second and the fourth and fifth toes) and shortened or absent middle phalanges, cardiac and renal malformations, vertebral anomalies and deafness (22). Defining the targets of this miRNAs gene will give a deeper understanding of the pathophysiology and complex genetics of GSD.

Finally, PPIs enriched was introduced, ‘H-sapiens_Module_19’, which was the most statistically significant with the *P*-value of 1.83E-17 among all PPIs, was mainly involved in the collagen group (COL1A1, COL1A2, COL2A1, COL9A1, COL9A2, COL9A3, COL10A1, COL11A1 and COL11A2) (23), the matrix metalloproteinase group (MMP2, MMP9 and MMP13) (24) and the Fibrillin-TGFbeta receptor group (causing overgrowth syndromes, including FBN1, FBN2, TGFβ1 and TGFβ2) (25).

The above functional and enrichment analysis results, including classical genetic and epigenetic modifications, is consistent with previous findings that skeletal system development, appendage development, limb development, appendage morphogenesis and limb morphogenesis are related to genetic skeletal disorders.

### Mutation annotation

‘SkeletonGenetics’ performed the detailed annotation information of all mutations to facilitate the users to assess the regarding interest mutations. Firstly, the coordinates of variations, such as SNV, (FBN1, NM_000138 and c.5198G > A) or InDels, (COL2A1, NM_033150 and c.4234_4245del) were converted to the corresponding coordinates on the human reference genome GRC37/hg19 (chr15:48755305-48755305) and (chr12:4836 7202- 4836 7213) by UCSC Genome Browser (26) and the in-house perl program was used to convert coordinates from CDS to genome. Secondly, the general annotation of mutations, such as the effects on protein coding (frameshift, non-frameshift, non-synonymous, splicing, stopgain, stoploss, etc.), amino acid change and the location of the mutation (exonic, intronic, intergenic, region, etc.) were performed by ANNOVAR (27). Additionally, more detailed clinical information was provided about each entry, including PubMed ID, ethnicity, gender (male or female), age-of-onset (death, newborn, days, weeks, months, years) and hereditary mode. Another 27 databases or data sets were linked and annotated, such as seven quick links (NCBI, HGNC, MGI, OMIM, Ensembl, Vega and GeneCards), 15 functional prediction software, dbSNP (28) and 1000 Genomes Project (29), ESP, CG69, ExAC. Phenotype information extracted from the HPO databases and provided the OMIM ID, MGI ID, phenotype or syndrome name (such as Weill–Marchesani syndrome 2, dominant), phenotype description (related to search module phenotype button), cosmic70, clinvar_20150330 information, etc.

### Mutation spectrum and gene–disease network

The location of the gene mutation is biased, some of which are located in the 5′-UTR region, 3′-UTR region or the mutation-rich region, and some are distributed in single mutation sites, such as 1138G > A mutation in FGFR3 of achondroplasia (30) and 49G > A mutation in AKT1 of proteus syndrome (31), which accounted for 98% and 100% of the total number of mutations, respectively. Therefore, in order to facilitate the search for mutation information and statistics on the bias of the mutation position, we used scalable vector graphics (SVG) to visualize the mutation distribution in each GSD-gene for related syndromes, each simulated fonts including gene position information, gene name (number of exons, transcript ID), and encoded information, with different colors to represent different mutation effects or types, which presented a gene level overview of the summarized mutations. For example, the syndrome of achondrogenesis type 2 (ACG2; Langer-Saldino), can be expressed as chr12:48 367 189-48 398 104, Gene: COL2A1 (54 coding exons, NM_001844 or NM_033150) and mutations in exonic or intronic, the variations were more than once distinguished by different colors and fonts from those first identified. Besides, SVG was used to construct a graphical gene-disease network to provide the potential relations of GSD and skeletal-related genes for understanding the complex heterogeneity of GSD. Information about the gene–disease network includes the number of GSD genes, mutation information and different disease phenotypes. For example, the COL2A1 related to nine common genetic skeletal disorders, including achondrogenesis type 2 (ACG2; Langer-Saldino), platyspondylic dysplasia, Torrance type, hypochondrogenesis, spondyloepiphyseal dysplasia congenital (SEDC), spondyloepimetaphyseal dysplasia (SEMD) strudwick type, kniest dysplasia, spondyloperipheral dysplasia, mild SED with premature onset arthrosis, SED with metatarsal shortening (formerly Czech dysplasia), stickler syndrome type 1, are depicted by a simple ball (red ball represents gene and blue ball represents disease) and a straight line to construct the gene–disease network. Users click on the corresponding graphics and can quickly link to detailed information on mutations and phenotypes.

### Data search and browse

**Figure 2.**
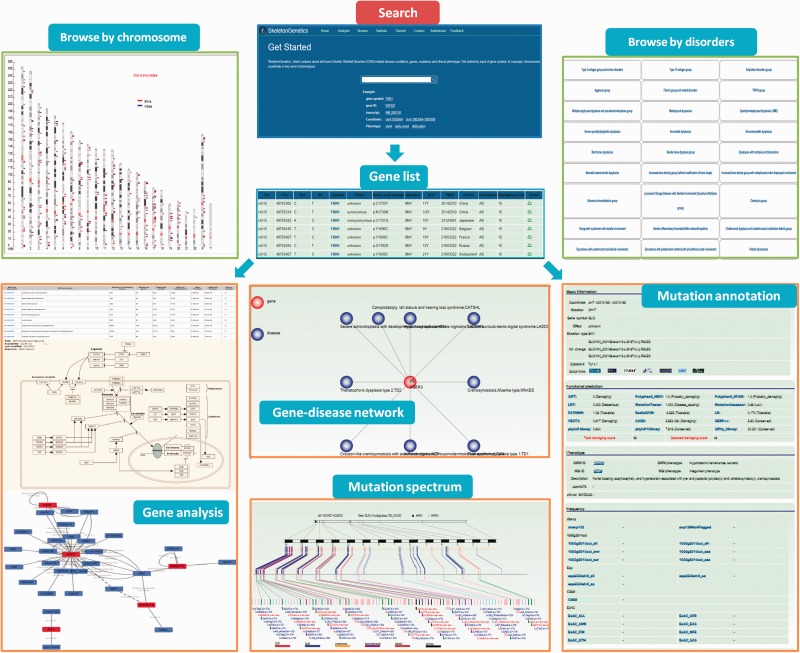
A screenshot of the search, browse and annotation module in ‘SkeletonGenetics’. Search box at home page for searching by five symbols. ‘gene symbol’, ‘gene ID’ and ‘gene transcript’, ‘mutations position’ and phenotype information. ‘Browse by chromosome’ is used to retrieve all GSD-related genes mapped on chromosomes, ‘Browse by disease’ is used to retrieve all GSD-related genes’. Annotation module including functional and enrichment analysis, mutation annotation, mutation spectrum and gene–disease network.

‘SkeletonGenetics’ provides a quick and concise search box on the home page for searching by five symbols. Firstly, there are three gene symbol search modules, ‘gene symbol’, ‘gene ID’ and ‘gene transcript’; secondly, mutation position and phenotype information were incorporated to allows users to search by (i) specifying options like gene name (capital letters), gene ID, transcript information (ii) investigating the specified phenotype of the typical skeletal changes related to the position of the forearm of the upper limb, to the description of the variability to the extent of skeletal changes (iii) search mutation position data of more than one gene or mutation, or when it is known that a mutation or gene is located on a particular area or chromosome, a position symbol list will be needed to achieve this fuzzy search. To facilitate users browsing the data, two different approaches are provided: (i) browse by disorders (ii) browse by chromosome (Figure 2). The ‘browse by disorders’ option provides 42 groups and 481 conditions of genetic skeletal disorders for users to conveniently retrieve the information about mutations of interest. The genes and mutations related to this group or disease conditions can be easily retrieved by selecting from the list. Additionally, users can browse all the variants that are mapped on the entry chromosome or chromosomal bands in a graphical way in ‘browse by chromosome’, which is linked to the mutations information page.

### Database organization and web interface

In ‘SkeletonGenetics’, all the data were stored and managed in a MySQL relational database and run on an Apache HTTP server by PHP and JavaScript program to provide a user-friendly web interface for searching and browsing. Meanwhile, the database was organized in two different table output formats by ANNOVAR software (27) and GOs annotation, KEGG pathways, Wikipathways, MicroRNA Target and Protein–Protein interaction were stored in separate tables. All data can be freely downloaded from the website (Download page). The web client has been successfully tested with Internet Explorer 10, Chrome 48.0, Safari 7.1 and Firefox 2/3 and is implemented independently of the operating system.

## Results and discussion

The assignment of genetic skeletal disorders into specific groups has been practiced since the previous versions of the ‘Nosology’ by biochemical, molecular information available, or the group of disorders with similar phenotypic features defined by common radiographic features or anatomical site. Meanwhile, researchers criticize the previous versions, focusing on their ‘hybrid’ nature, which does not stick to a single systematic approach. Firstly, disorders should be classified on phenotypic similarities. Secondly, they should be reclassified based on the pathway or gene related to the functional abnormality (4). Based on the above principles, and as more and more resources are published on the network resource, we developed a comprehensive database for genes and mutations related to genetic skeletal disorders, ‘SkeletonGenetics’ integrated data types associated with GSD through in-depth mining of 1698 publications and extensive functional analysis, which covered a broad range of data including lists of disease grouping and disease names, genes, mutations and mutations spectrum, GO terms, pathways, microRNA target, protein–protein interaction and gene–disease network. Meanwhile, combined with concise search methods, intuitive graphical displays, convenient browsing functions and constantly updatable features, the ‘SkeletonGenetics’ database could serve as a reclassified reference tool and valuable resource for unveiling the genetic and pathway basis of GSD.

With the development of the high-throughput sequencing, massive genetic skeletal disorder related genes and mutations have been identified in the past decade, but there is still about 30–40% of GSD with undiscovered disease genes (4, 6) because of the restriction of patients and complexity of the gene interaction network. In this study, functional analysis of the known GSD genes in publications mostly involved in specific GO items, such as skeletal system development, appendage development, limb development, appendage morphogenesis and limb morphogenesis or pathways, such as classical FGFs, TGF-beta, Hedgehog, WNT, Notch signaling pathways. Meanwhile, many of the new identified genes interact with known GSD genes (32–35) or key GSD genes could induce the disease state. In SkeletonGenetics, if researchers have found a specific GSD gene, they may link to the ‘Analysis’ page to see whether the new gene is involved in any skeletal-related GO terms, pathways or PPIs. This database would be important and useful for revealing novel GSD-related genes and pathways. Therefore, researchers could focus on the other unknown genes, which were involved in the same skeletal development and homeostasis functions module combined with biochemical, molecular information or the group of disorders with similar phenotypic features.

The increasing availability of massive parallel sequencing and other new sequencing technologies will likely result in a rapid and cost-effective identification of many GSD-causing genes and mutations, or novel phenotypes associated with mutations in genes already linked to other phenotypes. Automatic mining methods will be used in ‘SkeletonGenetics’ for updating GSD-related data, (1) collect the latest disorders, genes or mutation from PubMed or open databases related to GSD; (2) perform more extensive functional and enrichment analysis based on the updated data sets; (3) improve the mutation spectrum, gene–disease network and other database functionalities. In SkeletonGenetics, researchers may use the ‘Submission’ page to upload de novo mutations for GSD or new genetic skeletal disorders to keep the database up-to-date and comprehensive. We believe that ‘SkeletonGenetics’ will hopefully have paved the way by setting standards for the recognition and definition of skeletal phenotypes and understanding of the complex heterogeneity of GSD and hope that the continued efforts to improve ‘SkeletonGenetics’ will ultimately help improve diagnosis and treatment of genetic skeletal disorders.

## Supplementary data

Supplementary data are available at *Database* Online.

Supplementary Data
