# Supplementary material for: Skeleton Genetics: a comprehensive database for genes and mutations related to genetic skeletal disorders
Source: Database (Oxford). 2016 Aug 31;2016:baw127. doi: 10.1093/database/baw127 (PMC5006089; doi:10.1093/database/baw127)
Supplement: Supplementary Data [file supp_2016_baw127_index.html]

Skeleton Genetics: a comprehensive database for genes and mutations related to genetic skeletal disorders — Supplementary Data 

# Skeleton Genetics: a comprehensive database for genes and mutations related to genetic skeletal disorders

## Supplementary Data

files

- Supplementary Data - docx file
