# Supplementary material for: Skeleton Genetics: a comprehensive database for genes and mutations related to genetic skeletal disorders
Source: Database (Oxford). 2016 Aug 31;2016:baw127. doi: 10.1093/database/baw127 (PMC5006089; doi:10.1093/database/baw127)
Supplement: Supplementary Data [file supp_baw127_Supplement_Table_1.docx]

**Supplement Table 1. The grouping results and classification criteria of Genetic skeletal disorders**

| No. | Group name | Disease(No.) | Gene  (No.) | classification standard |
| --- | --- | --- | --- | --- |
| 1 | FGFR3 chondrodysplasia group | 6 | 1 | underlying gene or pathway |
| 2 | Type 2 collagen group | 11 | 4 | underlying gene or pathway |
| 3 | Type 11 collagen group | 5 | 2 | underlying gene or pathway |
| 4 | Sulphation disorders group | 8 | 6 | underlying gene or pathway |
| 5 | Perlecan group | 2 | 1 | underlying gene or pathway |
| 6 | Aggrecan group | 3 | 1 | underlying gene or pathway |
| 7 | Filamin group and related disorders | 10 | 3 | underlying gene or pathway |
| 8 | TRPV4 group | 5 | 1 | underlying gene or pathway |
| 9 | Ciliopathies with major skeletal involvement | 22 | 22 | localization of radiographic changes to specific bone structures (vertebrae, epiphyses, metaphyses, diaphysis, or thereof) or of the involved segment (rhizo, meso or acro) |
| 10 | Multiple epiphyseal dysplasia and pseudoachondroplasia group | 9 | 7 | localization of radiographic changes to specific bone structures (vertebrae, epiphyses, metaphyses, diaphysis, or thereof) or of the involved segment (rhizo, meso or acro) |
| 11 | Metaphyseal dysplasias | 9 | 7 | localization of radiographic changes to specific bone structures (vertebrae, epiphyses, metaphyses, diaphysis, or thereof) or of the involved segment (rhizo, meso or acro) |
| 12 | Spondylometaphyseal dysplasias (SMD) | 3 | 2 | localization of radiographic changes to specific bone structures (vertebrae, epiphyses, metaphyses, diaphysis, or thereof) or of the involved segment (rhizo, meso or acro) |
| 13 | Spondylo-epi-(meta)-physeal dysplasias (SE(M)D) | 10 | 9 | localization of radiographic changes to specific bone structures (vertebrae, epiphyses, metaphyses, diaphysis, or thereof) or of the involved segment (rhizo, meso or acro) |
| 14 | Severe spondylodysplastic dysplasias | 6 | 6 | localization of radiographic changes to specific bone structures (vertebrae, epiphyses, metaphyses, diaphysis, or thereof) or of the involved segment (rhizo, meso or acro) |
| 15 | Acromelic dysplasias | 16 | 13 | localization of radiographic changes to specific bone structures (vertebrae, epiphyses, metaphyses, diaphysis, or thereof) or of the involved segment (rhizo, meso or acro) |
| 16 | Acromesomelic dysplasias | 4 | 3 | localization of radiographic changes to specific bone structures (vertebrae, epiphyses, metaphyses, diaphysis, or thereof) or of the involved segment (rhizo, meso or acro) |
| 17 | Mesomelic and rhizo-mesomelic dysplasias | 8 | 7 | localization of radiographic changes to specific bone structures (vertebrae, epiphyses, metaphyses, diaphysis, or thereof) or of the involved segment (rhizo, meso or acro) |
| 18 | Campomelic dysplasia and related disorders | 2 | 2 | macroscopic criteria in combination with clinical features (bent bones, slender bones, presence of multiple dislocations). |
| 19 | Slender bone dysplasia group | 21 | 20 | macroscopic criteria in combination with clinical features (bent bones, slender bones, presence of multiple dislocations). |
| 20 | Dysplasias with multiple joint dislocations | 4 | 4 | macroscopic criteria in combination with clinical features (bent bones, slender bones, presence of multiple dislocations). |
| 21 | Chondrodysplasia punctata (CDP) group | 9 | 8 | increased or reduced bone density, impaired mineralization, stippling, osteolysis |
| 22 | Neonatal osteosclerotic dysplasias | 4 | 4 | increased or reduced bone density, impaired mineralization, stippling, osteolysis |
| 23 | Osteopetrosis and related disorders | 19 | 17 | osteopetrosis (OP) variants and related disorders |
| 24 | Other sclerosing bone disorders | 16 | 12 | increased or reduced bone density, impaired mineralization, stippling, osteolysis |
| 25 | Osteogenesis Imperfecta and decreased bone density group | 35 | 26 | osteogenesis imperfecta and decreased bone density group |
| 26 | Abnormal mineralization group | 13 | 11 | hypophosphatemic rickets |
| 27 | Lysosomal Storage Diseases with Skeletal Involvement (Dysostosis Multiplex group) | 24 | 21 | lysosomal disorders with skeletal involvement |
| 28 | Osteolysis group | 6 | 5 | increased or reduced bone density, impaired mineralization, stippling, osteolysis |
| 29 | Disorganized development of skeletal components group | 17 | 15 | abnormal development of skeletal components such as exostoses, ecnhondromas, and ectopic calcification |
| 30 | Overgrowth syndromes with skeletal involvement | 13 | 12 | overgrowth syndromes with significant skeletal involvement |
| 31 | Genetic inflammatory/rheumatoid-like osteoarthropathies | 8 | 8 | genetic inflammatory/rheumatoid-like osteoarthropathies |
| 32 | Cleidocranial dysplasia and isolated cranial ossification defects group | 4 | 4 | dysostoses and follow again anatomical criteria (cranium, face, axial skeleton, extremities) with additional criteria reflecting principles of embryonic development |
| 33 | Craniosynostosis syndromes | 18 | 12 | dysostoses and follow again anatomical criteria (cranium, face, axial skeleton, extremities) with additional criteria reflecting principles of embryonic development |
| 34 | Dysostoses with predominant craniofacial involvement | 13 | 13 | dysostoses and follow again anatomical criteria (cranium, face, axial skeleton, extremities) with additional criteria reflecting principles of embryonic development |
| 34 | Dysostoses with predominant vertebral with and without costal involvement | 14 | 14 | dysostoses and follow again anatomical criteria (cranium, face, axial skeleton, extremities) with additional criteria reflecting principles of embryonic development |
| 36 | Patellar dysostoses | 8 | 8 | dysostoses and follow again anatomical criteria (cranium, face, axial skeleton, extremities) with additional criteria reflecting principles of embryonic development |
| 37 | Brachydactylies (without extraskeletal manifestations) | 8 | 7 | dysostoses and follow again anatomical criteria (cranium, face, axial skeleton, extremities) with additional criteria reflecting principles of embryonic development |
| 38 | Brachydactylies (with extraskeletal manifestations) | 17 | 17 | dysostoses and follow again anatomical criteria (cranium, face, axial skeleton, extremities) with additional criteria reflecting principles of embryonic development |
| 39 | Limb hypoplasia–reduction defects group | 33 | 30 | dysostoses and follow again anatomical criteria (cranium, face, axial skeleton, extremities) with additional criteria reflecting principles of embryonic development |
| 40 | Ectrodactyly with and without other manifestations | 11 | 8 | dysostoses and follow again anatomical criteria (cranium, face, axial skeleton, extremities) with additional criteria reflecting principles of embryonic development |
| 41 | Polydactyly-Syndactyly-Triphalangism group | 27 | 23 | dysostoses and follow again anatomical criteria (cranium, face, axial skeleton, extremities) with additional criteria reflecting principles of embryonic development |
| 42 | Defects in joint formation and synostoses | 5 | 3 | dysostoses and follow again anatomical criteria (cranium, face, axial skeleton, extremities) with additional criteria reflecting principles of embryonic development |
